# Supplementary material for: A Comprehensive Assessment of Ultraviolet-Radiation-Induced Mutations in Flammulina filiformis Using Whole-Genome Resequencing
Source: J Fungi (Basel). 2024 Mar 20;10(3):228. doi: 10.3390/jof10030228 (PMC10971301; doi:10.3390/jof10030228)
Supplement: Supplementary file 1 [file jof-10-00228-s001.zip › Supplementary Material S8/KEGG annotation/out/64381550635650.os/KO/out_map/map05014.html]

KEGG PATHWAY: Amyotrophic lateral sclerosis (ALS) - Reference pathway


|  |  |
| --- | --- |
| **Amyotrophic lateral sclerosis (ALS) - Reference pathway** |  |

[
Pathway menu
| Organism menu
| Pathway entry
| Show description
| User data mapping
]

Amyotrophic lateral sclerosis (ALS) is a progressive, lethal, degenerative disorder of motor neurons. The hallmark of this disease is the selective death of motor neurons in the brain and spinal cord, leading to paralysis of voluntary muscles. Mutant superoxide dismutase 1 (SOD1), as seen in some familial ALS (FALS) cases, is unstable, forming aggregates in the motor neuron cytoplasm, axoplasm and mitochondria. Within mitochondria, mutant SOD1 may interfere with the anti-apoptotic function of Bcl-2, affect mitochondrial import by interfering with the translocation machinery (TOM/TIM), and generate toxic free radicals (ROS). Reactive oxygen species (ROS), produced within mitochondria, inhibit the function of EAAT2, the main glial glutamate transporter protein, responsible for most of the reuptake of synaptically released glutamate. Glutamate excess increases intracellular calcium, which enhances oxidative stress and mitochondrial damage. Mutant SOD1 can also trigger oxidative reactions , which can then cause damage through the formation of hydroxyl radicals or via nitration of tyrosine residues on proteins. Nitration may target neurofilament proteins, affecting axonal transport. Collectively, these mechanisms are predicted to disturb cellular homeostasis, ultimately triggering motor neuron death.

|  |  |
| --- | --- |
| Reference pathway | 100% |
